# Survival Advantage Comparing Older Living Donor Versus Standard Criteria Donor Kidney Transplants

**DOI:** 10.3389/ti.2024.12559

**Published:** 2024-03-11

**Authors:** Kamlesh Patel, Anna Brotherton, Daoud Chaudhry, Felicity Evison, Thomas Nieto, Dilan Dabare, Adnan Sharif

**Affiliations:** ^1^ Department of Nephrology and Transplantation, University Hospitals Birmingham, Birmingham, United Kingdom; ^2^ School of Medical and Dental Sciences, University of Birmingham, Birmingham, United Kingdom; ^3^ Data Science Team, Research Development and Innovation, University Hospitals Birmingham, Birmingham, United Kingdom; ^4^ Institute of Immunology and Immunotherapy, University of Birmingham, Birmingham, United Kingdom

**Keywords:** kidney transplantation, mortality, survival, older living donor, standard criteria donor

## Abstract

The aim of this analysis was to explore mortality outcomes for kidney transplant candidates receiving older living donor kidneys (age ≥60 years) versus younger deceased donors or remaining on dialysis. From 2000 to 2019, all patients on dialysis listed for their first kidney-alone transplant were included in a retrospective cohort analysis of UK transplant registry data. The primary outcome was all-cause mortality, with survival analysis conducted by intention-to-treat principle. Time-to-death from listing was modelled using nonproportional hazard Cox regression models with transplantation handled as a time-dependent covariate. A total of 32,978 waitlisted kidney failure patients formed the primary study cohort, of whom 18,796 (58.5%) received a kidney transplant (1,557 older living donor kidneys and 18,062 standard criteria donor kidneys). Older living donor kidney transplantation constituted only 17.0% of all living donor kidney transplant activity (overall cohort; *n* = 9,140). Recipients of older living donor kidneys had reduced all-cause mortality compared to receiving SCD kidneys (HR 0.904, 95% CI 0.845–0.967, *p* = 0.003) and much lower all-cause mortality versus remaining on the waiting list (HR 0.160, 95% CI 0.149–0.172, *p* < 0.001). Older living kidney donors should be actively explored to expand the living donor kidney pool and are an excellent treatment option for waitlisted kidney transplant candidates.

## Graphical Abstract

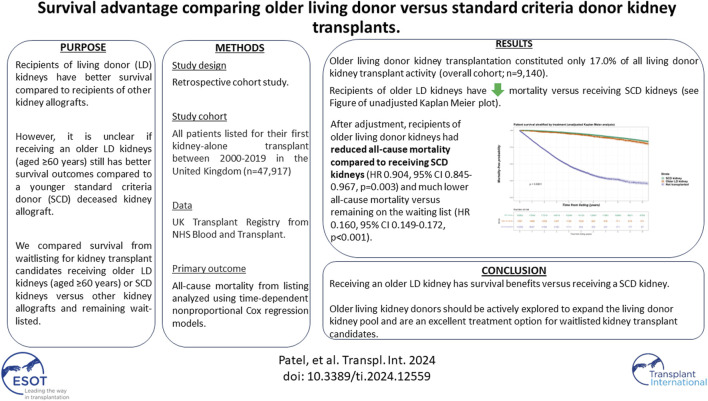

## Introduction

Living donor kidney transplantation is the optimal treatment of choice for kidney failure patients deemed suitable for surgery. In a systematic review and meta-analysis of 48 published cohort studies, any recipient of a living donor kidney had superior all-cause mortality compared to recipients of other kidney allografts or remaining waitlisted on dialysis [1]. This mirrors national registry data, with superior ten-year patient and graft survival reported after living donor kidney transplantation versus deceased donor kidney transplantation [2].

Despite these benefits, living donor kidney transplant rates have stagnated over the last decade in many kidney transplant programs. In the United Kingdom, living donor transplant rates have dropped by a quarter over the last decade, from a peak of 1,036 adult living donor kidney transplants in the year 2013/2014 to 789 in the last available year of 2021/2022 [3]. While some of this may relate to recovery processes post pandemic, it is notable that living donor kidney transplant rates pre-pandemic in 2019/2020 were only 954. Therefore, a key component of the latest NHS Blood and Transplant (NHSBT) strategy document encourages expansion of living donor kidney transplantation activity [4]. To that effect, promoting living kidney donation among older individuals is very attractive. Bailey et al. report the number of living kidney donors aged ≥65 years has risen from 4% to 10% between 2006 and 2017 respectively [5]. However, numbers appear to have plateaued since then. According to national registry data, while 18% of all living donor kidney donors were aged ≥60 years between 2010 and 2016 [6], this has remained static at 20% between 2016 and 2022 [3].

The literature regarding survival outcomes for kidney transplant candidates receiving older living donor kidneys is not clear. In a systematic review of published studies, living donor age stratified at 60 years was associated with 1-year graft loss for recipients but no significant findings were observed for either 1- or 3-year recipient mortality or graft loss at a lower donor age stratification of 50 years [7]. However, the meta-analysis for mortality was conducted on three small studies for publications between 1989 and 2010, which severely limits its utility and interpretation. Other work has associated older living donor age as a risk factor for graft loss and/or mortality when compared to a younger living donor [8, 9]. However, this is not a useful comparison as many kidney transplant candidates will not have a choice between an older or younger living donor. More relevant is whether survival outcomes differ when comparing receipt of an older living donor kidney versus receiving a standard criteria donor (SCD) kidney. This is an important question which kidney transplant candidates may be faced in the real-world and there is a paucity of contemporary literature to guide counselling on this matter. Therefore, the aim of this analysis was to explore this question using UK transplant registry data, with older living donors defined as any donor aged 60 years and above.

## Materials and Methods

### Study Cohort

A retrospective cohort study was undertaken of prospectively collected registry data related to all waitlisted kidney failure patients receiving dialysis in the United Kingdom. From January 1, 2000 until September 30, 2019 inclusive, all patients who were either listed and received a first kidney-alone transplant in the United Kingdom versus those who were listed but never received a kidney transplant were included in the study. No formal sample size estimate was conducted as all eligible patient records were used. December 31, 2020 was considered the study end. The study is reported as per STROBE guidance [10].

### Study Variables

The following study variables were available for all patients; age (at listing and at transplantation), sex, ethnicity (classified as white, black, Asian [Indo-Asian], other, known), primary cause of kidney failure (classified as diabetes, glomerulonephritis, hypertension, other separate, polycystic kidney disease, pyelonephritis/reflux nephropathy, unknown/missing), year of listing, and waiting time.

Donor kidneys were stratified into living donors (with older living donors defined at an age ≥60 years) or SCD. Donors after brain and circulatory death (DBD and DCD respectively) were handled the same way. The primary cohort was obtained by excluding any expanded criteria donor (ECD) kidney recipients from the deceased donor cohort if they fulfilled the following criteria: 1) deceased donor aged ≥60 years, or 2) deceased donor aged between 50 and 59 years with any two from the following three additional criteria; hypertension; raised creatinine and/or death from stroke). However, secondary analyses were conducted with the inclusion of ECD kidney transplant recipients. The remaining waitlisted kidney transplant candidates did not proceed for transplantation and remained on dialysis.

### Outcomes

The primary outcome of interest was all-cause mortality. The survival analysis was conducted according to the intention-to-treat principle; therefore, patients were not dropped from the analysis if they were removed from the waiting list or if transplantation subsequently failed. Secondary outcomes explored include death-censored graft loss.

### Statistical Analysis

For baseline demographics, continuous variables were reported as medians and interquartile ranges (IQRs) and compared between groups using Mann-Whitney tests. Ordinal factors were also compared using Mann-Whitney tests, whilst nominal factors were analysed using Fisher’s exact tests or Chi-square tests for those with two or more than two categories, respectively. Missing data underwent list-wise deletion and complete case analysis was undertaken.

Survival was analysed as time from initial placement on the waiting list to death, with data censored at loss of follow up or on December 31, 2020. Unadjusted survival-free probability was analysed by generation of Kaplan–Meier curves. After testing for violations of the proportional hazard assumption, time-to-death was modelled using nonproportional hazard Cox regression models with transplantation handled as a time-dependent covariate. Using this approach, all patients contribute data for time at risk (and death if it occurs) to the non-transplant group starting at study entry before some switch and contribute time at risk (and death if it occurs) to the transplant group starting at the time of transplantation (this forms the time-dependent transplant covariate in the model). Mortality hazard ratios were computed for the transplant recipients compared with those on the waiting list. We explored adjusted models factoring for age, sex, ethnicity, cause of kidney failure and year of placement on the waiting list. Time to graft loss models were conducted using survival/censoring-weighted Cox regression models and adjusted for age at listing, sex, ethnicity, cause of kidney failure, year of placement of the waiting list, level of HLA mismatches, delayed graft function and 1-year rejection.

Due to heterogenous statistical methods used for reported transplant studies, as reported in Supplementary Table D from the systematic review by Chaudhry et al. [1], complementary survival analyses were undertaken to investigate the robustness of our primary model. These included; 1) survival/censoring-weighted Cox regression, which is a parsimonious alternative to a standard Cox regression model and provides interpretable average effects in the either the presence or absence of non-proportional hazards [11], 2) re-analysis to overcome immortality bias by comparing time from transplant versus time from waitlisting for transplant versus non-transplant cohorts respectively, 3) weighted Cox regression of a propensity score matched cohort after nearest neighbour 1:1 matching (for age at listing, sex, ethnicity, cause of kidney failure and waiting time), and 4) extended nonproportional hazard Cox regression model with transplantation and graft loss handled as a time-dependent variables. Furthermore, subgroup analyses with different older living donor age stratifications were undertaken versus both SCD and ECD kidneys.

All analyses were done using R 4.0.4 (R Foundation for Statistical Computing, Vienna, Austria), with packages including *coxphw* (survival analyses) [11] and *MatchIt* (propensity-score matching).

### Approvals

National Health Service Blood and Transplant (NHSBT) in the United Kingdom obtains informed consent from all patients undergoing solid organ transplantation for data collection and subsequent analyses. Study proposals are reviewed and approved by the kidney advisory group on behalf of NHSBT before data dissemination.

## Results

### Study Cohort

**FIGURE 1:**
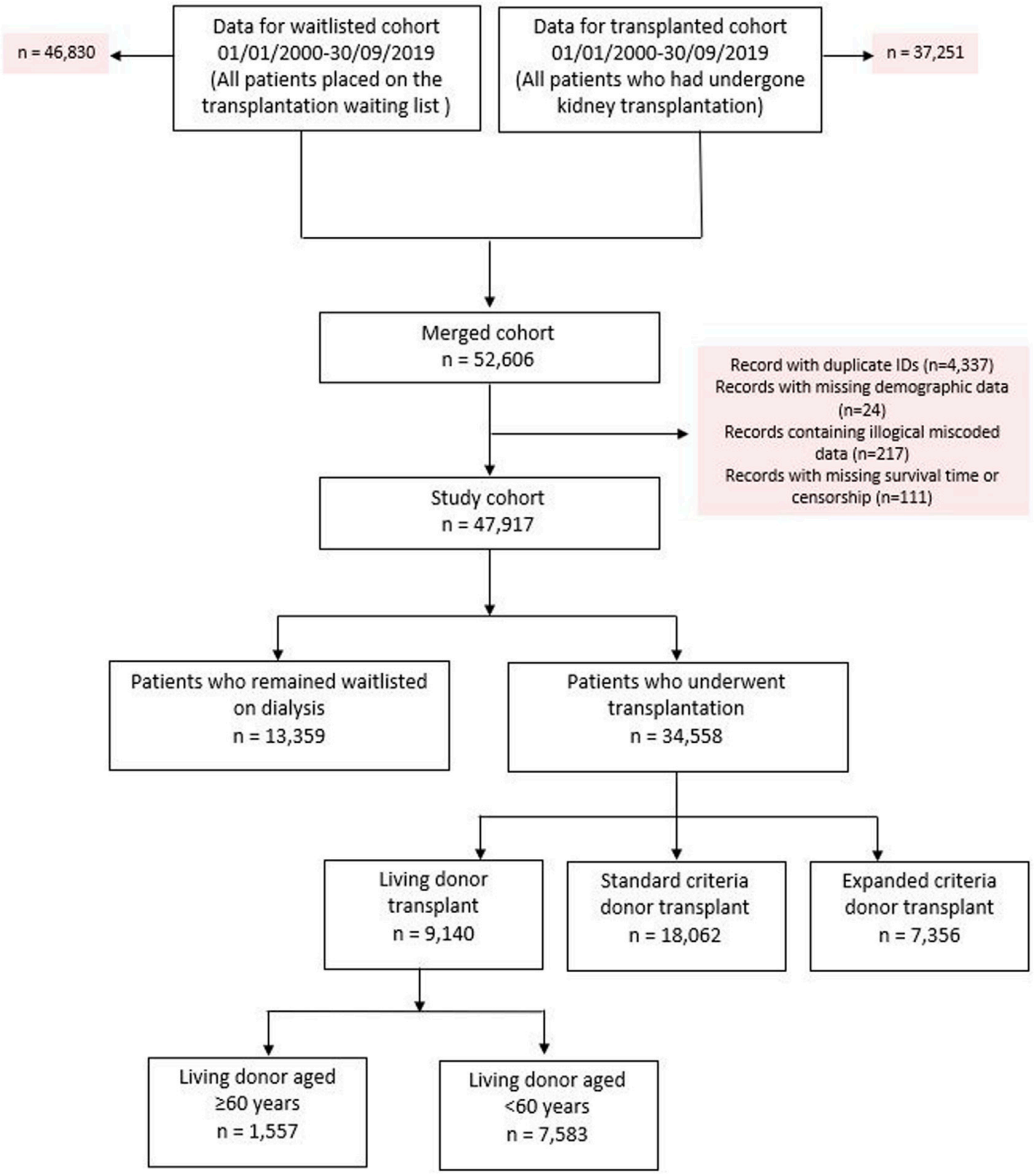
PRISMA flowchart of study cohort.

The original cohort obtained from NHSBT contained records from two datasets between January 1, 2000 until September 30, 2019; kidney failure patients listed who received a kidney transplant (*n* = 37,251) and kidney failure patients listed for transplantation (*n* = 46,830). After combining both datasets, duplicated records and/or cases with missing demographic data were excluded. This left 47,917 kidney failure patients to form our total study cohort, of whom 34,558 (72.1%) subsequently received their first kidney transplant after waitlisting (living donors; *n* = 9,140, SCD; *n* = 18,062 and ECD; *n* = 7,356). For the primary analysis, we excluded recipients of ECD and living donor kidneys aged <60 years (*n* = 7,583), which left a primary study cohort of 32,978. Observation time for the study cohort involved a total of 222,896 patient-years, with median follow up 5.8 years. See Figure 1 for the PRISMA flowchart.

Table 1 shows baseline demographics at the time of listing for the study cohort and identifies significant differences in baseline demographics between those that received different types of kidney allografts versus those that remained without transplantation. Table 2 compares waitlisted kidney transplant candidates who received older versus younger living donors, showing very different demographics between the recipients of both kidneys. Supplementary Figure S1 shows the evolution of age demographics among living kidney donors over the study cohort period, highlighting the increase in proportion of living donor donors aged ≥60 years from the beginning of the study period but static percentages in recent years.

**TABLE 1:** Baseline demographics of waitlisted kidney failure patients.

Variable		LD kidney	SCD kidney	ECD kidney	Dialysis	*p*-Value
Percentage (n)		19.1% (9,140)	37.7% (18,062)	15.4% (7,356)	27.9% (13,359)	—
Median Age at waitlisting in years (IQR)		43 (23)	45 (19)	57 (15)	53 (21)	<0.001
Sex	Male	61.4% (5,611)	62.7% (11,326)	64.2% (4,719)	61.0% (8,143)	<0.001
	Female	38.6% (3,529)	37.3% (6,736)	35.8% (2,637)	39.0% (5,216)	
Ethnicity	White	82.6% (7,550)	75.3% (13,593)	75.2% (5,532)	71.6% (9,564)	<0.001
	Asian	8.8% (808)	13.4% (2,418)	13.5% (990)	15.5% (2,072)	
	Black	4.8% (436)	7.7% (1,383)	7.5% (554)	9.0% (1,198)	
	Other	2.8% (252)	2.7% (496)	3.0% (219)	3.1% (416)	
	Unknown	1.0% (94)	1.0% (172)	0.8% (61)	0.8% (109)	
Cause of kidney failure	Diabetes	7.2% (659)	7.5% (1,351)	12.3% (903)	27.6% (3,681)	<0.001
	Glomerulonephritis	6.6% (602)	6.8% (1,231)	6.3% (462)	3.8% (511)	
	Hypertension	4.7% (431)	5.3% (950)	6.7% (491)	4.7% (633)	
	Other Separate	31.8% (2,905)	27.2% (4,911)	24.7% (1,815)	20.9% (2,787)	
	Polycystic Kidney	8.9% (810)	11.5% (2,072)	12.4% (909)	6.3% (845)	
	Pyelonephritis/reflux	6.9% (629)	7.8% (1,411)	5.9% (431)	4.4% (592)	
	Unknown/Missing	34.0% (3,104)	34.0% (6,136)	31.9% (2,345)	32.3% (4,310)	
Waiting time in days (IQR)		230 (576)	791 (1,016)	896 (988)	475 (614)	<0.001

LD, living donor; SCD, standard criteria donor; ECD, expanded criteria donor; IQR, interquartile range.

**TABLE 2:** Characteristics of recipient receiving living donor kidneys.

Recipient variables		All LD kidney	Old LD (aged ≥60 years)	Young (aged <60 years)	*p*-Value
Percentage (*n*)		100.0% (9,140)	17.0% (1,557)	83.0% (7,580)	—
Median Age at waitlisting in years (IQR)		43 (23)	51 (25)	42 (22)	<0.001
Median Age at transplantation in years (IQR)		44 (23)	53 (26)	45 (22)	<0.001
Sex	Male	61.4% (5,611)	60.4% (940)	61.6% (4,669)	0.366
	Female	38.6% (3,529)	39.6% (617)	38.4% (2,911)	
Ethnicity	White	82.6% (7,550)	87.5% (1,363)	81.6% (6,184)	<0.001
	Asian	8.8% (808)	6.6% (102)	9.3% (706)	
	Black	4.8% (436)	2.6% (40)	5.2% (396)	
	Other	2.8% (252)	2.3% (36)	2.8% (216)	
	Unknown	1.0% (94)	1.0% (16)	1.0% (78)	
Cause of kidney failure	Diabetes	7.2% (659)	8.5% (133)	6.9% (526)	<0.001
	Glomerulonephritis	6.6% (602)	6.4% (99)	6.6% (503)	
	Hypertension	4.7% (431)	5.3% (83)	4.6% (348)	
	Other Separate	31.8% (2,905)	32.8% (510)	31.6% (2,395)	
	Polycystic Kidney	8.9% (810)	11.0% (171)	8.4% (638)	
	Pyelonephritis/reflux	6.9% (629)	6.2% (96)	7.0% (533)	
	Unknown/Missing	34.0% (3,104)	29.9% (465)	34.8% (2,637)	
Waiting time in days (IQR)		230 (576)	275 (632)	223 (558)	<0.001
Time period	2010 onwards	4,330	52.8% (822)	46.3% (3,507)	<0.001
	Pre 2010	4,808	47.2% (735)	53.7% (4,073)	

LD, living donor; IQR, interquartile range.

### Mortality Events

In the primary study cohort, waitlisted kidney failure patients who did not receive kidney transplants had 4,003 deaths (30.0% of dialysis cohort) versus 3,

701 deaths in the SCD group (20.5% of cohort) versus 257 deaths in the older living donor group (16.5% of older living donor cohort).

**FIGURE 2:**
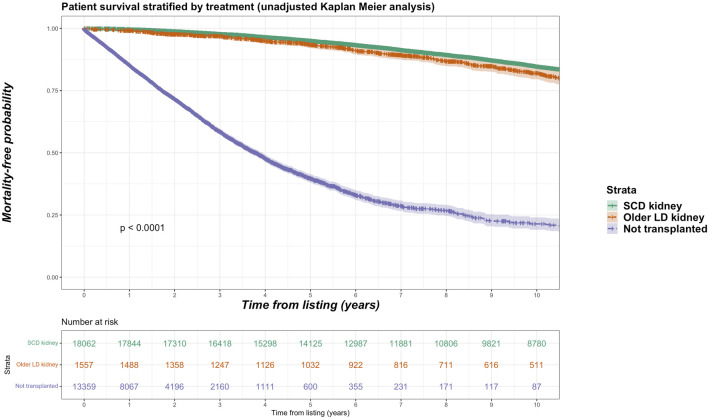
Unadjusted Kaplan-Meir plot of mortality free survival comparing recipients of older living donor kidneys versus standard criteria kidneys versus remaining waitlisted on dialysis from listing.

**FIGURE 3:**
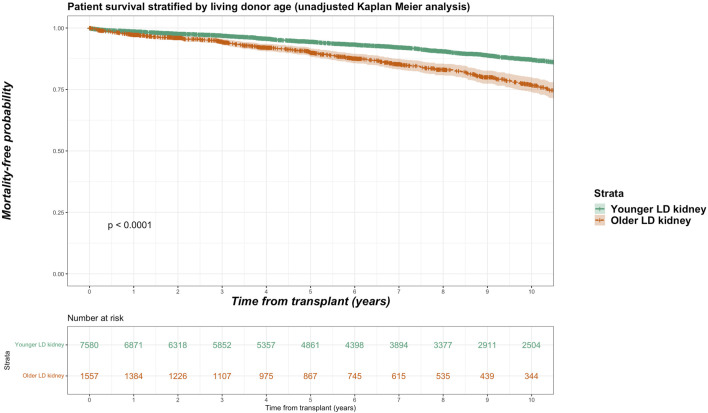
Unadjusted Kaplan-Meir plot of mortality free survival comparing recipients of older versus younger living donor kidneys from listing.

For the living donor transplant group, 257 deaths in the older living donor cohort compares with 870 deaths (11.5% of total deaths) of the younger living donor cohort. Unadjusted Kaplan-Meir plot for mortality stratified by older living donor kidneys versus alternative treatments from listing is shown in Figure 2, while in Figure 3 an unadjusted mortality comparison is made between older versus younger living donor kidney transplants from surgery.

### Unadjusted and Adjusted Graft Survival (Death-Censored) Using Weighted Cox Regression

**FIGURE 4:**
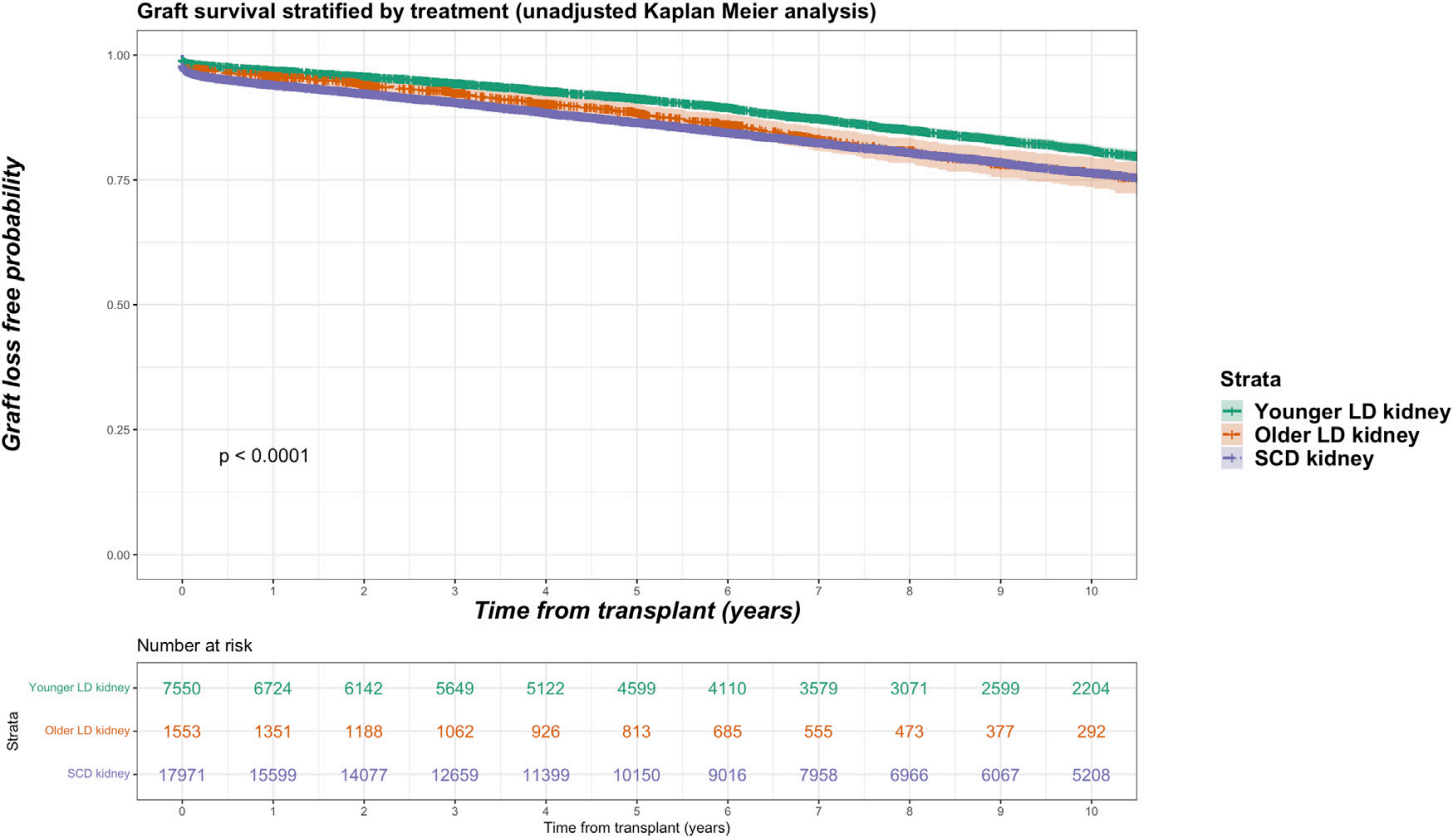
Unadjusted Kaplan-Meir plot of graft loss free survival comparing recipients of older living donor kidneys versus younger living donor kidneys versus standard criteria kidneys from transplant.

Death-censored graft losses over the follow up period were compared between older living kidney, younger living kidney and SCD kidney transplant recipients. Overall, 3,658 graft losses occurred in the SCD cohort (20.4% of SCD group) versus 249 graft losses in the older living donor cohort (16.0% of older living donor group). In younger living donor kidney recipients, a total of 1,189 graft losses occurred (15.7% of younger living donor group). Unadjusted Kaplan-Meir plots for death-censored graft loss stratified by older living donor, younger living donor and SCD kidneys is shown in Figure 4.

In adjusted models, compared to receiving a SCD kidney, receiving an older living donor kidney was associated reduced risk for graft loss (HR 0.872, 95% CI 0.761–1.000, *p* = 0.050) independent of other variables. No significant difference in risk for graft loss was observed comparing older to younger living donor kidneys (HR 1.273, 95% CI 0.956–1.695, *p* = 0.098).

### Adjusted Mortality Analyses

#### Nonproportional Hazards Cox Regression Model With Transplantation a Time-dependent Covariate

In a non-proportional hazard Cox regression model using a time-dependent analysis, with transplantation handled as a time-dependent covariate, recipients of older living donor kidneys had reduced all-cause mortality compared to receiving SCD kidneys (HR 0.904, 95% CI 0.845–0.967, *p* = 0.003) and much lower all-cause mortality versus remaining on the waiting list (HR 0.160, 95% CI 0.149–0.172, *p* < 0.001) independent of other variables. We conducted a non-proportional Cox regression analysis with both transplantation and graft loss factored as time-dependent covariates. In this extended model, receiving older living kidneys still had reduced risk for all-cause mortality versus receiving SCD kidneys (HR 0.897, 95% CI 0.851–0.946, <0.001) or remaining on dialysis (HR 0.149, 95% CI 0.141–0.158, *p* < 0.001) independent of other variables. This is shown in Table 3.

**TABLE 3:** Non-proportional hazard Cox model of predictors for mortality after kidney transplantation with either dialysis or SCD as reference [fully adjusted model with transplantation (Model 1) or transplantation + graft loss (Model 2) handled as a time varying covariate].

Variable		HR (95% CI)	Variable		HR (95% CI)
Treatment (Model 1)	Dialysis	1.000	Treatment	SCD	1.000
	SCD	0.177 (0.171–0.184)		Dialysis	5.641 (5.445–5.844)
	LD	0.160 (0.149–0.172)		LD	0.904 (0.845–0.967)
Treatment (Model 2)	Dialysis	1.000	Treatment	SCD	1.000
	SCD	0.166 (0.161–0.172)		Dialysis	6.021 (5.827–6.221)
	LD	0.149 (0.141–0.158)		LD	0.897 (0.851–0.946)

LD, living donor; SCD, standard criteria donor; HR, hazard ratio; CI, confidence interval.

#### Alternate Survival Models

**FIGURE 5:**
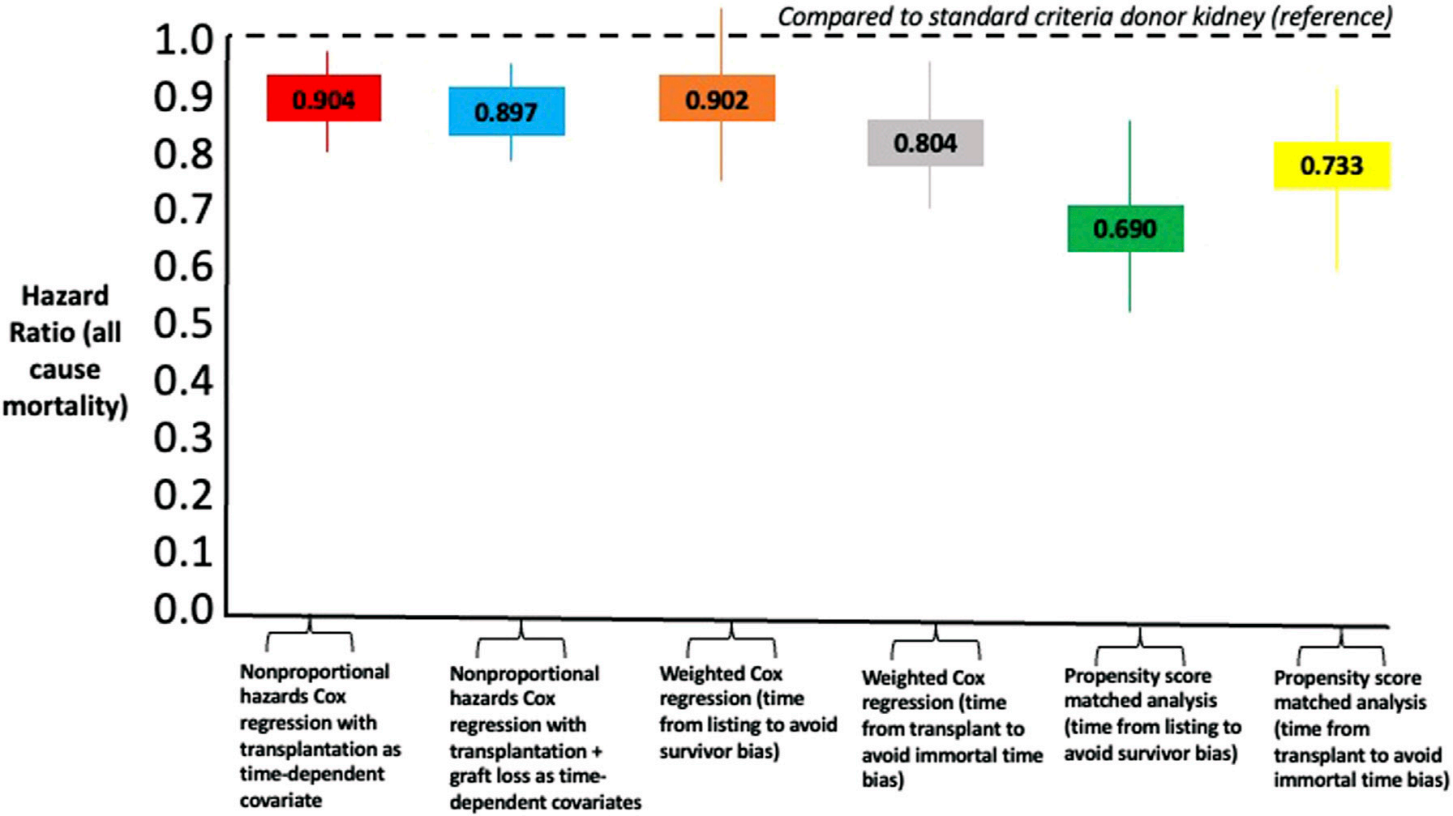
Comparison of Hazard Ratios for all-cause mortality using different statistical models comparing recipients of older living donor kidneys versus standard criteria kidneys as reference point.

In a survival/censoring-weighted Cox regression model, compared to SCD kidney recipients, older living donor kidney recipients had equivalent all-cause mortality after waitlisting (Hazard Ratio 0.902, 95% CI 0.774–1.051, *p* = 0.187) but lower all-cause mortality compared to dialysis (HR 0.100, 05% CI 0.085–0.118, *p* < 0.001). In a model that overcomes immortal time bias for pre-transplant survival on the waiting list, recipients of older living donor kidneys had lower all-cause mortality compared to SCD kidneys (HR 0.804, 95% CI 0.701–0.923, *p* = 0.002) versus remaining on the waiting list (HR 0.163, 95% CI 0.141–0.189, *p* < 0.001). In a propensity score matched cohort comparing older living donor kidney recipients with SCD (balance plot shown in Supplementary Material S2), older living donor kidney recipients had reduced all-cause mortality from listing (HR 0.690, 95% CI 0.547–0.872) or from transplant (HR 0.733, 95% CI 0.0.597–0.899, *p* = 0.003). Figure 5 summarizes the comparative Hazard ratios from the different models.

#### Sub-analyses (Older Living Donor Age Stratified by Age Groups)

**FIGURE 6:**
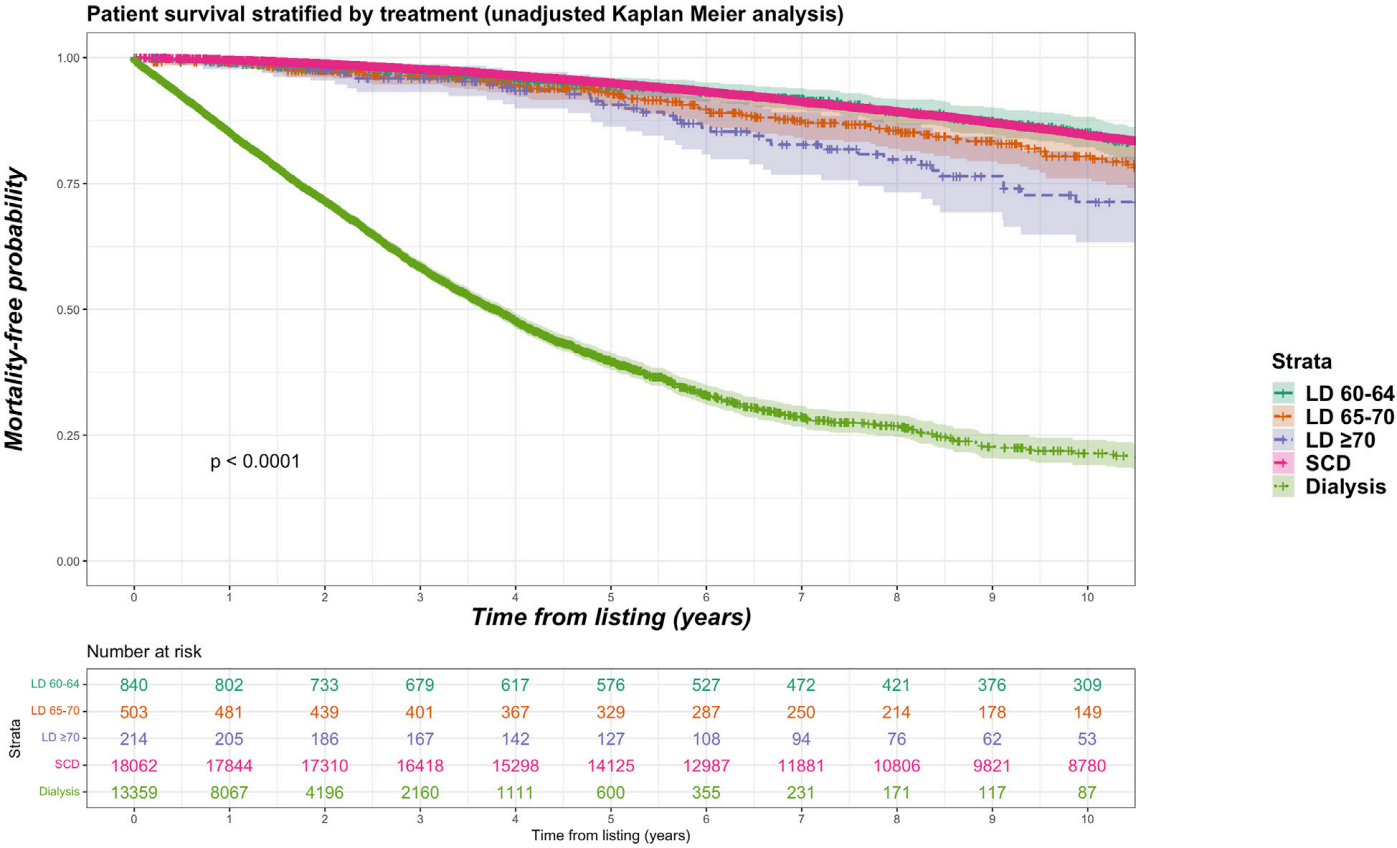
Unadjusted Kaplan-Meir plot of mortality free survival comparing recipients of older living donor kidneys stratified by age groups (60–64 years, 65–69 years, ≥70 years) versus standard criteria donor kidneys versus remaining waitlisted on dialysis from listing.

**FIGURE 7:**
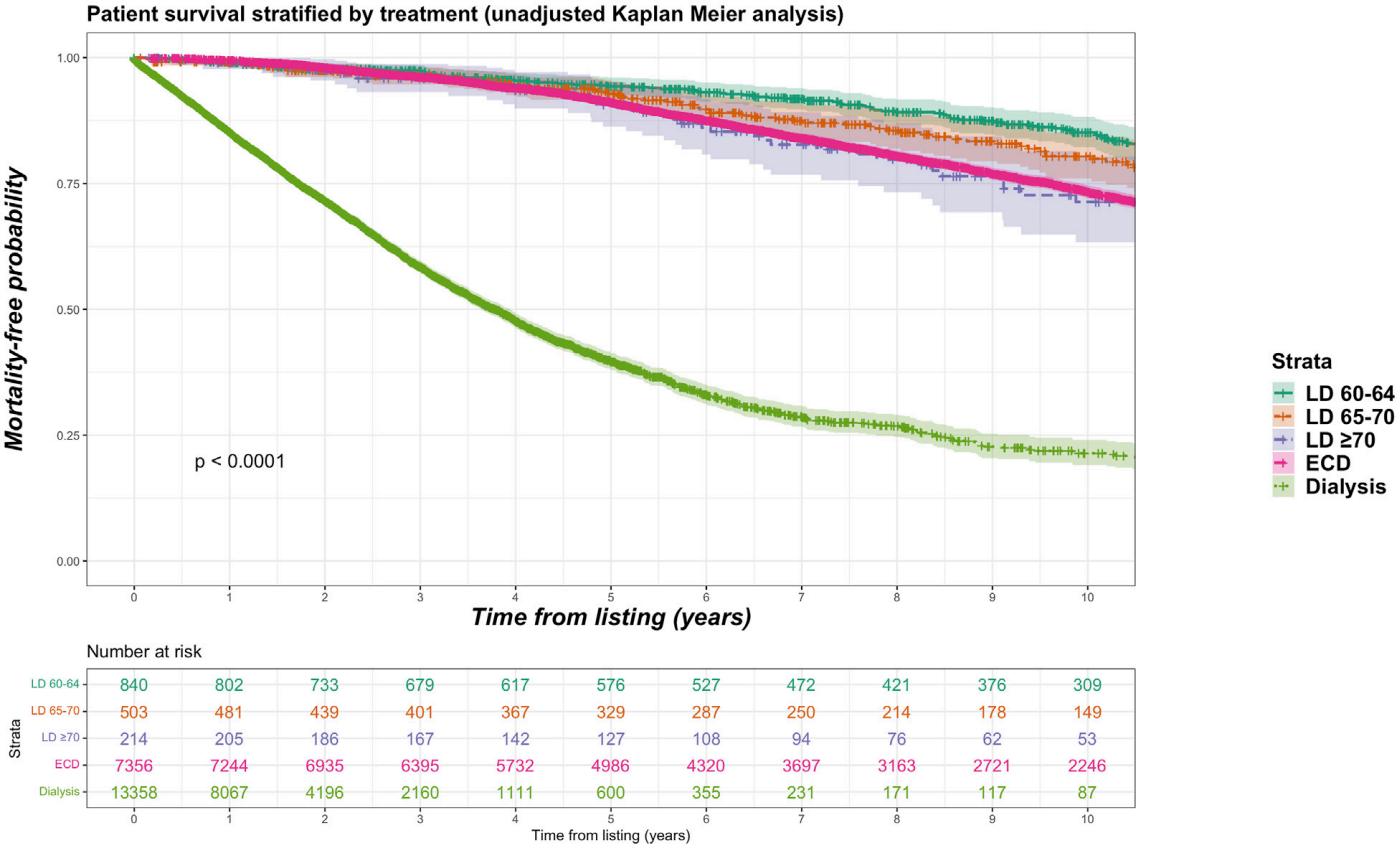
Unadjusted Kaplan-Meir plot of mortality free survival comparing recipients of older living donor kidneys stratified by age groups (60–64 years, 65–69 years, ≥70 years) versus expanded criteria donor kidneys versus remaining waitlisted on dialysis from listing.

Sub-group analyses were undertaken with different stratifications for older living donor age. We identified 840 living donors aged between 60 and 64 years (9.2% of total living donor cohort, median 61 years), 503 living donors aged between 65 and 70 (5.5% of total living donor cohort, median 66 years) and 214 donors aged 70 years and over (2.3% of total living donor cohort, median 72 years). Mortality rate was 15.2%, 16.7% and 21.0% for recipients of kidneys from living donor age groups 60–64, 65–69 and ≥70 years respectively. Figure 6 shows unadjusted Kaplan-Meier plots of all-cause mortality for recipients of the different older living donor age groups versus SCD kidneys versus remaining on dialysis. Figure 7 shows unadjusted Kaplan-Meier plots of all-cause mortality for recipients of the different older living donor age groups versus ECD kidneys versus remaining on dialysis.

Table 4 summarizes the output from a non-proportional time-dependent hazard Cox regression model, with transplantation handled as a time-dependent covariate, comparing all-cause mortality for recipients of older living kidney stratified by age groups. The comparator is versus SCD or ECD kidney transplant recipients, with remaining on dialysis also included. In comparison to receiving a SCD kidney, recipients of older living donor kidneys from anyone aged 60–64 years or 65–69 years had lower all-cause mortality while higher all-cause mortality was observed for recipients of living donor kidneys aged ≥70 years. In comparison to receiving a ECD kidney, recipients of older living donor kidneys from anyone aged 60–64 years or 65–69 years had lower all-cause mortality but equivalent all-cause mortality was observed for recipients of living donor kidneys aged ≥70 years.

**TABLE 4:** Non-proportional hazard Cox model of predictors for mortality after kidney transplantation with SCD or ECD as reference (fully adjusted model with transplantation handled as a time varying covariate).

Variable		Hazard ratio (95% CI)	
		SCD as reference	ECD as reference
Treatment	LD aged 60–64	0.857 (0.782–0.940)	0.697 (0.634–0.765)
	LD aged 65–69	0.857 (0.762–0.963)	0.724 (0.644–0.815)
	LD aged ≥70	1.232 (1.052–1.443)	1.066 (0.910–1.249)
	Dialysis	5.646 (5.450–5.850)	4.811 (4.628–5.001)
Median Age at waitlisting in years (IQR)		1.044 (1.043–1.045)	1.033 (1.031–1.034)
Sex	Female	REF	REF
	Male	1.111 (1.081–1.143)	1.230 (1.187–1.275)
Ethnicity	White	REF	REF
	Asian	0.797 (0.764–0.833)	0.758 (0.720–0.799)
	Black	0.754 (0.711–0.800)	0.698 (0.639–0.739)
	Other	0.637 (0.572–0.710)	0.676 (0.600–0.762)
	Unknown	0.992 (0.873–1.127)	1.128 (0.916–1.388)
Cause of kidney failure	Diabetes	REF	REF
	Glomerulonephritis	0.426 (0.400–0.454)	0.434 (0.400–0.470)
	Hypertension	0.504 (0.471–0.539)	0.461 (0.424–0.501)
	Other Separate	0.446 (0.426–0.467)	0.456 (0.431–0.481)
	Polycystic Kidney	0.376 (0.355–0.397)	0.405 (0.378–0.433)
	Pyelonephritis/reflux	0.508 (0.478–0.540)	0.514 (0.475–0.555)
	Unknown/Missing	0.479 (0.460–0.500)	0.487 (0.464–0.512)
Year of listing		0.926 (0.923–0.929)	0.940 (0.937–0.943)

LD, living donor; SCD, standard criteria donor; ECD, expanded criteria kidney; CI, confidence interval.

## Discussion

The literature reports heterogenous outcomes for recipients of older living donor kidneys, dependent upon whether comparisons are made with different types of deceased donor allografts or younger living donors. From a practical perspective, the key question is whether waitlisted kidney transplants candidates likely to receive SCD kidneys have any survival advantage or disadvantage to proceed with an older living kidney donor versus a SCD kidney. In our contemporary population cohort study, our findings suggest receiving an older living donor kidney (aged ≥60 years) is associated with lower mortality and risk of graft loss versus receiving an SCD kidney. On sensitivity analyses with older living donor age stratified, all older living donor age groups provide a mortality benefit except receiving a kidney from a living donor aged ≥70 years, which was associated with higher mortality compared to receiving a SCD kidney (but equivalent mortality when compared to receiving an ECD kidney).

Disparate outcomes from previous studies reflect era effects, variable definitions, diverse study populations, methodological differences, and different study comparators (e.g., recipients of younger living donor or SCD kidneys). Favorable outcomes are reported in a 1990–2010 cohort from the United States, where 219/97,782 (0.2%) of all living kidney donors were identified as aged older than ≥70 years [9]. No statistically significant difference in recipient survival was seen between those who received kidneys from living kidney donors aged ≥70 years versus matched recipients of kidneys from younger living kidney donors aged 50–59 years (HR 1.31, 95% CI 0.95–1.69). When compared to matched recipients of SCD kidneys from deceased donors aged 50–59 years, no statistically significant difference in patient survival was seen (HR 0.79, 95% CI 0.60–1.03) [9]. Although not statistically significant, the effect sizes are clinically significant and likely to reflect type 2 statistical errors in view of low sample size. A subsequent registry analysis using data from the United Network for Organ Sharing (UNOS) dataset between 1994 and 2012 was undertaken by Englem et al., with 4.4% of the living donor cohort (4,186/92,646) aged ≥60 years (3.2% aged 60–64 years; 1.0% aged 65–69 years; 0.2% aged ≥70 years) [12]. Compared to SCD recipients, no difference in overall graft survival was observed between living donors aged 65 years or older but risk for death-censored graft loss was higher. Transplant recipients with older living donor kidneys had significantly lower graft and overall survival compared to younger living donor recipients.

Examining a contemporary cohort is important, as era effects may be present. Iordanous et al. identified inferior patient and graft survival for recipients of older (aged 60–85 years) versus younger (aged 30–55 years) living donor kidneys in a systematic review and meta-analysis of study cohorts published between 1980 and 2008, although survival differences dissipated in the 2000s [13]. In subsequent work by the same group using data from Ontario, Canada between 2000 and 2008, no significantly increased risk for death (HR 1.83, 95% CI 0.96–3.48, *p* = 0.07) or graft-censored graft loss (HR 0.71, 95% CI 0.32–1.56, *p* = 0.39) was observed with median follow up 4 years for older living kidney donors (aged ≥60 years) versus SCD kidney recipients [14]. However, the hazard ratio was not proportional and increased with time, meaning uncertainty for longer outcomes. This is consistent with data from the Scientific Registry of Transplant Recipients (SRTR), which demonstrate 10-year adjusted hazard ratios for death or graft loss among recipients increase in a non-linear fashion with increasing living donor age and is highest among the ≥60 years group (compared to the reference of living donors aged 18–30 years) [15].

When compared to published data, our results provide reassurance that older living kidney donors provide a survival advantage for kidney transplant candidates versus receiving a SCD (but survival disadvantage if the living donor is aged ≥70 years). For candidates more likely to receive ECD kidneys, there is survival advantage using an older living kidney donor (and survival equivalence if the living donor is aged ≥70 years). Utilization of living donors aged ≥70 years, while a small proportion of the overall living donor cohort, requires careful matching of donors and recipients to facilitate optimized outcomes. One suggestion is to avoid extreme age differences when considering living donors aged ≥70 years. In a small single-center study, Hiramitsu et al. observed living donor kidney transplantation from donors aged 70–89 years to recipients with a donor-recipient age difference of 10–15 years was an independent risk factor for graft loss and recipient mortality [8]. This complements our analyses and suggest living kidney donors aged ≥70 years are an appropriate choice for kidney transplant candidates likely to receive ECD kidneys but not SCD kidneys (or any candidate if compared to dialysis).

This is an important and topical question, especially as countries strive to expand living donor numbers. In the United States, data from the SRTR show living kidney donors aged ≥55 years have been the fastest growing cohort among all living kidney donor activity and are now the second commonest age group between 40 and 54 years (which has been slowly declining) [16]. If living donor activity can successfully increase, especially among older adults as potential donors, then our data can influence decision making for optimized patient counselling. Parallel to discussions about recipient survival outcomes are the safety outcomes associated with using older living kidney donors. Although low among living kidney donors, cumulative 15-year incidence of end-stage kidney disease per 10,000 varies significantly by age and is highest for donors aged ≥60 years 70.2 (95% CI 30.4–161.8, *p* < 0.001) [17]. Some of this risk may be due to an age-related sluggish physiological response by the contralateral kidney after donor nephrectomy. In a retrospective single-center analysis, Bellini et al. observed slower recovery of kidney function for living donors aged ≥60 years and higher percentual difference in estimated glomerular filtration rate (eGFR) post-donation [18]. This was consistent with findings from a systematic review and meta-analysis of 31 published studies [19]. While low eGFR is an independent risk factor for cardiovascular disease and all-cause mortality, any increased risk for these outcomes has reassuringly not been observed among older living donors. Analyzing UNOS data, Segev et al. observed no increase in mortality for living kidney donors versus age-matched “healthy” nondonors when stratified by age [3,017/80,347 (3.8%) living donors were aged ≥60 years] [20]. Reese et al., specifically matched older living kidney donors (mean age 59 years) from the UNOS dataset to healthy older individuals in the Health and Retirement Study, finding no difference in risk for cardiovascular disease or death. In summary, older age *per se* should not be considered a contra-indication to being a living kidney donor [21]. However, rigorous selection criteria is warranted and careful donor-recipient matching necessary for optimized outcomes.

Our study has many strengths in comparison to the available published literature. Firstly, our cohort of 2000–2019 is more contemporary than previous studies, reflecting current clinical practice and selection criteria. Many allocation systems aim to match like-for-like for donors and recipients like the United Kingdom, which should make these results translatable to other countries with similar allocation policies. Secondly, we have utilized different statistical approaches to test for robustness. It is reassuring to observe the take-home messages from our analyses are generally consistent across all statistical models used and reinforces our primary study findings. Limitations of this study must also be appreciated for accurate interpretation of the results. As an intention-to-treat analysis, we did not factor for waitlisted kidney failure patients who were suspended or removed from the waiting list due to lack of fitness. This could lead to informative-treatment bias, i.e., where the pool of transplants recipients is systematically different from the remaining-on-dialysis comparator group. Censoring patients at delisting would have yielded an overestimation of survival on dialysis as data from the United Kingdom confirms increased mortality associated for waitlisted kidney failure patients who experience any period of suspension [22]. This analysis comprised waitlisted kidney transplant candidates who either had their primary transplant or remained on dialysis; therefore it provides no targeted evidence in the setting of advanced chronic kidney disease or a failed kidney transplant exploring repeat transplantation. Lack of data relating to medical co-morbidities or dialysis vintage limits interpretation of survival probabilities in the setting of specific health burdens, which may tip the balance of more borderline risk versus benefit calculations for recipients of older living kidney donors. Residual confounding is an important but inevitable limitation of retrospective registry analyses despite adjusted statistical analyses. This is certainly the case in this analysis due to unavailable data and unmeasured confounders. Finally, this analysis has focused solely upon survival benefits associated with transplant surgery for kidney failure patients and overlooks the importance of quality of life which was beyond the scope of this study but is under investigation elsewhere [23].

To conclude, in this contemporary national cohort study of kidney failure patients listed for transplantation, proceeding with an older living donor kidney transplant affords a survival benefit to kidney transplant candidates when compared to receiving a standard criteria donor kidney or remaining on dialysis. While our data is reassuring, the caveat remains that survival benefits at a population-level must be translated to individual kidney transplant candidates with personalized risk counselling (e.g., using living donors aged ≥70 years). However, our data provides reassurance to clinicians involved in the care of kidney failure patients that older living donor candidates are an untapped pool of potential kidney donors that should be actively pursued.

## Data Availability

Publicly available datasets were analyzed in this study. This data can be found here: UK transplant registry.
